# Tunable multiphase dynamics of arginine and lysine liquid condensates

**DOI:** 10.1038/s41467-020-18224-y

**Published:** 2020-09-15

**Authors:** Rachel S. Fisher, Shana Elbaum-Garfinkle

**Affiliations:** 1 Structural Biology Initiative, CUNY Advanced Science Research Center, New York, NY USA; 2 Ph.D. Programs in Biochemistry and Biology at the Graduate Center, City University of New York, New York, NY USA

**Keywords:** Biophysical chemistry, Biopolymers in vivo, Intrinsically disordered proteins, Biomaterials - cells

## Abstract

Liquid phase separation into two or more coexisting phases has emerged as a new paradigm for understanding subcellular organization, prebiotic life, and the origins of disease. The design principles underlying biomolecular phase separation have the potential to drive the development of novel liquid-based organelles and therapeutics, however, an understanding of how individual molecules contribute to emergent material properties, and approaches to directly manipulate phase dynamics are lacking. Here, using microrheology, we demonstrate that droplets of poly-arginine coassembled with mono/polynucleotides have approximately 100 fold greater viscosity than comparable lysine droplets, both of which can be finer tuned by polymer length. We find that these amino acid-level differences can drive the formation of coexisting immiscible phases with tunable formation kinetics and can be further exploited to trigger the controlled release of droplet components. Together, this work provides a novel mechanism for leveraging sequence-level components in order to regulate droplet dynamics and multiphase coexistence.

## Abstract (web summary)

The design principles underlying biomolecular phase separation of membrane-less organelles remain poorly understood. Using model homopolymers, Fisher et al. show that the formation kinetics of coexisting liquid phases can be tuned by exploiting differences between arginine and lysine residues.

## Introduction

Liquid–liquid phase separation of biomolecules has emerged as a ubiquitous driving force underlying subcellular organization, from modern cells to the protocellular origins of life^1–3^. Coacervation of proteins and nucleic acids into liquid droplets, increasingly referred to as “biomolecular condensates”^1^, has been implicated in the assembly of membraneless organelles^4–6^, in the coordination of genetic elements^7–9^ and cytoskeletal regulatory molecules^10,11^, and in the etiology of diseases from cancer to neurodegeneration^2,12–14^. The collective emergent material properties of condensates and their regulation have been underscored as essential features of condensate function and/or dysfunction^2,14–17^. Deciphering the mechanism underlying the assembly of individual biomolecules into condensates with unique material properties and the interaction or coexistence between distinct phases impacts our understanding of current and past cellular life, human health, and additionally drives a new frontier toward the engineering of organelles with controllable and even novel functions^18–21^.

Current understanding of the biomolecular driving forces underlying liquid phase separation has been successfully informed by classic theories of model polymer coacervates^22,23^, including the role of length-dependent multivalent interactions^10,11^. The interplay of electrostatic, hydrophobic, and cation–pi interactions^22–27^ have been further demonstrated to contribute to the condensate interactome. Specific amino acids, such as arginine (R) and lysine (K), have been identified as key residues in driving phase separation in vitro and in vivo. Arginine residues are essential features of arginine/glycine (R/G)-rich domains^28^ that drive phase separation of the proteins DDX4^29^, LAF-1^30^, FUS^31,32^, FMRP^33^, Lsm4^34^, and PGL proteins^35,36^, and arginine methylation can regulate phase separation of these domains^29,33,37^. Similarly, lysine residues and their acetylation have been shown to be crucial for the liquid phase separation of the proteins tau^38^ and DDX3X^39^. Interestingly, despite the conserved charge between residues, arginine-to-lysine mutations in R/G-rich domains result in decreased phase separation propensity with higher critical concentrations required for phase separation^29,33^.  Additionally, the properties of arginine-rich and lysine-rich condensates exhibit significant differences.  Recent fluorescence recovery after photobleaching (FRAP) studies indicate that proline–arginine dipeptide repeats implicated in ALS give rise to condensates that have less internal mobility than comparable proline–lysine dipeptide repeats^40^, with similar observations of reduced fluidity made for model arginine–glycine vs lysine–glycine peptide sequences^41^ and lysine and arginine-rich peptides^42^. These recent works have additionally shown that substituting poly-RNA bases (purine vs pyrimidine) had distinct consequences on the apparent fluidity of respective arginine- vs lysine-rich peptides. Direct rheological measurements comparing arginine and lysine homopolymer condensates would provide fundamental insight into how these residues contribute to network properties, such as viscosity.

Material properties such as viscosity and surface tension dictate many essential characteristics of condensates, including internal diffusion rates, molecular sequestration, and the hierarchical organization of coexisting phases^43,44^. The coexistence of multiple phases has recently been demonstrated to play important roles in cellular function, including the organization of the nucleolus^45^, FMRP/CAPRIN1 droplets^46^, and P granule proteins^36^. The sequence-driven rules underlying the multiphase droplet formation of charged biopolymers are just beginning to be unraveled. Previous work has shown that the miscibility of distinct phases of hydrophobic elastin-like polypeptides can be regulated by sequence changes that alter the critical temperature of phase separation^47^. More recent works have shown that coexisting phases of charged polyelectrolytes can, when sufficiently different, form multiple phases^48,49^. Where there is a difference in critical salt concentration, which is indicative of a different density and water content between complex coacervates, multiple phases will form. Different homopolymeric RNAs, due to difference in cation–pi interaction strength between arginine and nucleobases, have also been shown to be sufficient in creating multiphase droplets^40^. These coexisting phases of charged polyelectrolytes can influence solute partitioning^48,49^ due in part to unique microenvironments brought about by relative density differences^49^. Despite these advances, it is not yet understood what differences on the amino acid residue level are sufficient to drive the formation of coexisting phases. In addition, recent work has been studied under equilibrium conditions, while the kinetic processes and directed manipulation of multiphase dynamics remains largely unexplored.

Here we set out to ask whether differences between lysine and arginine condensates could be exploited to regulate multiphase dynamics and stability. We find that the minimal nucleobase unit required for condensate formation differs between poly-L-lysine (polyK) and poly-L-arginine (polyR) sequences. Using microrheology to precisely quantify condensate viscosity, we show that arginine–nucleotide droplets have approximately 100-fold greater viscosity than comparable lysine–nucleotide condensates, which is a significantly larger difference than that observed when increasing polymer length (between *N* = 10 to *N* = 100). We find that lysine and arginine polymers are not miscible within condensates, and arginine antagonistically competes for anionic complexation. We demonstrate that the differences between droplets can be exploited to rapidly invert lysine-rich droplets inducing release of lysine polymers into the surrounding environment. Furthermore, by altering the stoichiometry and length of polymers and nucleotides, the rate of inversion and polymer release can be tuned allowing for coexisting phases to persist over varying timescales. This work utilizes the distinct phase behaviors of lysine and arginine residues, resulting from underlying differences in interaction, to offer a fundamental mechanism for the control and manipulation of droplet dynamics and multiphase coexistence.

## Results

### Polymer length tunes viscosity of polyK liquid condensates

**Fig. 1:**
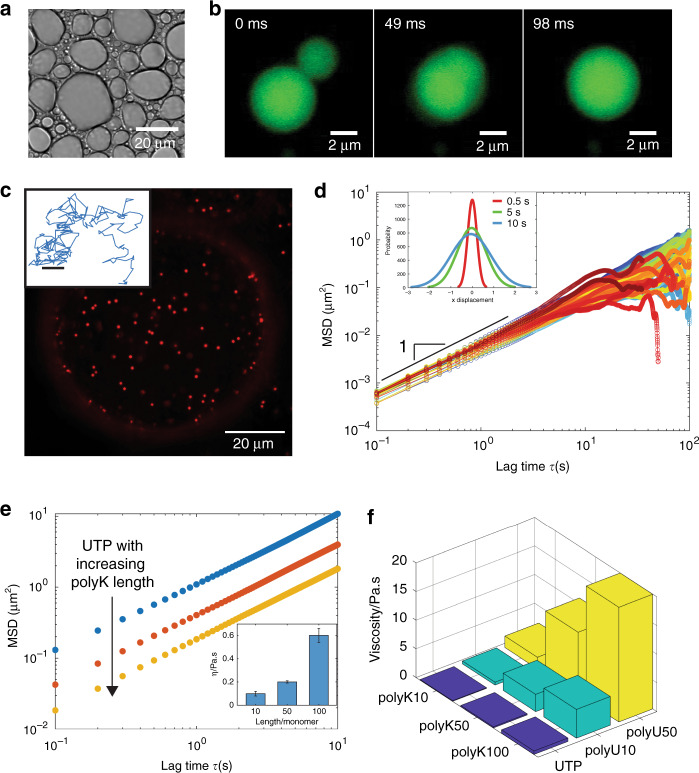
Viscosity of poly-L-lysine coacervates is controlled by polymer length. **a** Brightfield image of polyK100/UTP condensates (10 mM Tris, pH 7.4). polyK concentration 6 mM per monomer and uridine triphosphate (UTP) 1.5 mM per monomer. **b** Widefield fluorescence image of polyK100/UTP condensate fusion (partitioned free Atto488 dye incorporated for enhanced visualization). **c** Confocal fluorescence image of polyK100/UTP droplet with 500 nm beads embedded (Red FluoSpheres, Invitrogen). Inset, representative 2D bead track. Scale bar 0.1 μm **d** Mean squared displacement (MSD) vs lag time for individual 500 nm beads in polyK100/UTP droplets. Inset, distribution of bead displacements at lag times = 0.5 s (red), 5 s (green), 10 s (blue). **e** MSD data for polyK10 (blue), polyK50 (red), and polyK100 (yellow) with UTP. Inset, viscosity as a function of polyK length. **f** Viscosity vs polyK length for polymers with UTP (blue), pU10 (green), and pU50 (yellow).

In order to extract fundamental rules linking condensate molecular components to emergent material properties, we first examine the contribution of polymer length, using three different fixed lengths of polyK (*N* = 10, 50, 100) combined with uridine phosphates and poly-uridine (pU) (*N* = 10, 50). At a total concentration of 6 mM per monomer, all lengths of polyK form liquid droplets capable of rapid droplet fusion in complex with charge-matched quantities of pU and uridine-5’-triphosphate trisodium salt (UTP; Fig.1a, b and Supplementary Fig. 1), consistent with the coacervation of oppositely charged polymers (reviewed here^50,51^) and the more recently observed phase separation of mixed length polyK with mononucleoside triphosphates^52^, respectively. We find that polyK cannot, however, form droplets with uridine-5′-driphosphate disodium salt (UDP) or uridine-5′-monophosphate (UMP) under the conditions tested (Supplementary Fig. 1).

To quantify the viscoelasticity of the droplets, we utilized microrheology, a technique based on tracking the motion of fluorescent tracer beads embedded with in condensates (Fig. 1c) in order to obtain the mean-squared displacement (MSD),

1 $${\mathrm{MSD}}(\tau ) = \left\langle {\left( {{\mathbf{r}}(\tau + t) - {\mathbf{r}}{\mathrm{(}}{\it{t}}{\mathrm{)}}} \right)^2} \right\rangle.$$

A concentration of 6 mM per monomer of polyK and charge-matched polyanion concentration were used for all rheological measurements. Microbeads embedded within polyK condensates display Brownian motion with Gaussian displacement distributions, and fitting the MSD over time gives a diffusive exponent equal to 1 demonstrating that polyK condensates are pure viscous fluids with no elastic component (Fig. 1c, d). As polymer length increases, bead motion slows and there is a downward shift of the MSD. Using

2 $${\mathrm{MSD}} = 4Dt$$

and the Stokes–Einstein relation

3 $$D = \frac{{k_BT}}{{6\pi \eta R}},$$

where *D* = diffusion coefficient, *T* = temperature, *η* = viscosity, and *R* = bead radius, we determine the viscosity for polyK10-UTP droplets to be 0.1 Pa.s (similar viscosity to maple syrup). Droplet viscosity increases with increasing polyK length, with *η* = 0.2 and 0.6 for polyK50-UTP and polyK100-UTP, respectively (Fig. 1e and Table 1). polyK droplet viscosity increases further when complexed with pU10 and pU50 with the highest viscosity values increasing to approximately 20 Pa.s for the longest polymer complex, polyK100-pU50 (Fig. 1f and Table 1). Viscosity scales with polyK polymer length, *N*, for both the pU10 and pU50 condensates (Supplementary Fig. 2) suggesting an unentangled polymer solution according to the Rouse model^53^. Interestingly, the viscosity for polyK-UTP condensates appears to demonstrate a weaker dependence on polymer length than for pU10 or pU50 condensates, suggesting distinct modes of interaction for mononucleotides and polynucleotides (Supplementary Fig. 2).

**Table 1:** Coacervate viscosity.

# monomers	UDP		UTP		pU10		pU50	
	polyK	polyR	polyK	polyR	polyK	polyR	polyK	polyR
10	‒	6.5 (±0.3)	0.1 (±0.02)	36 (±2.3)	0.5 (±0.07)	53 (±2)	2 (±0.4)	198 (±34)
50	‒	13 (±1.3)	0.2 (±0.01)	65 (±2)	3 (±0.7)	118 (±4.6)	11 (±0.5)	>280
100	‒	41 (±11.3)	0.6 (±0.06)	‒	5 (±0.2)	235 (±13.4)	20 (±1.1)	>280

Viscosities (Pa.s) for all combinations of polyK and polyR with UDP/UTP/pU, which form coacervates. Standard deviation is derived from three individual experiments at 19 ± 2 °C.

### Arginine and lysine polymers exhibit distinct phase behavior

Recent work in the field of biological phase separation has highlighted important roles for lysine^38,54^ and arginine^29,55–58^ residues. Moreover, despite the conserved charge between residues, arginine-to-lysine mutations in R/G-rich domains result in decreased phase separation propensity with higher critical concentrations required for droplet formation^29,33^. We therefore sought to quantify the differences in assembly propensity and material properties of arginine/lysine homopolymer condensates.

**Fig. 2:**
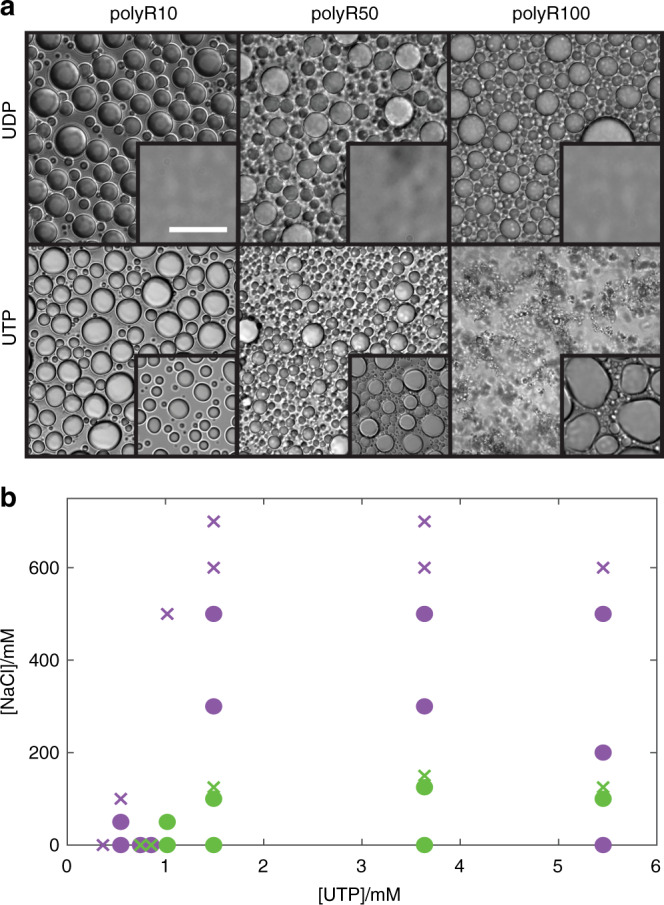
Differences in assembly propensity of polyR and polyK droplets. **a** DIC images showing (i) polyR10-UDP, polyR50-UDP, and polyR100-UDP (10 mM Tris, pH 7.4) with insets displaying polyK under same condition and (ii) polyR10-UTP, polyR50-UTP, and polyR100-UTP (10 mM Tris, pH 7.4). Concentration polyK/polyR 6 mM per monomer, UTP 1.5 mM, UDP 2 mM. Scale bar 20 μm. **b** Phase diagram for polyK50 (green) and polyR50 (purple) (6 mM per monomer) with varying NaCl and UTP concentrations. Green circles denote conditions under which polyK50-UTP droplet formation is observable. Purple circles denote conditions where polyR50-UTP droplet formation is observable.

We find that, under identical conditions, arginine polymers display differences in propensities for phase separation when compared to lysine analogs. Whereas all polyK lengths tested form droplets with UTP, only polyR10-UTP and polyR50-UTP form droplets, while polyR100-UTP assembles into amorphous aggregates (Fig. 2a and Supplementary Fig. 3). In addition, while polyK polymers are unable to coacervate with UDP, all polyR lengths tested do form droplets with UDP (Fig. 2a). To determine whether these differences arise from differences in relative interaction strengths, we constructed a phase diagram as a function of NaCl concentration for polyR50-UTP and polyK50-UTP. We find a significant shift in droplet stability as a function of increasing NaCl concentration, with polyR droplets persisting at higher salt at a given polymer/UTP concentration (Fig. 2b). To further examine differences in interaction strength between these two polymers and uridine, we used fluorescence correlation spectroscopy (FCS) to measure binding affinity. Using pU10-alexa488 (50 nM) and increasing amounts of polyK10, we determine a binding affinity of 6.5 μM for polyK/pU. Interestingly, extracting a dissociation constant for polyR10 was not possible, as binding was concomitant with phase separation, even at sub-μM concentrations (Supplementary Fig. 4). In contrast, no phase separation was observed for polyK up to 1 mM concentrations tested. Thus arginine and lysine display inherent differences in binding strength with identical partners (Fig. 2b), as well as unique modes of interaction with distinct partners (Fig. 2a).

### polyR droplets are over 100× more viscous than polyK

**Fig. 3:**
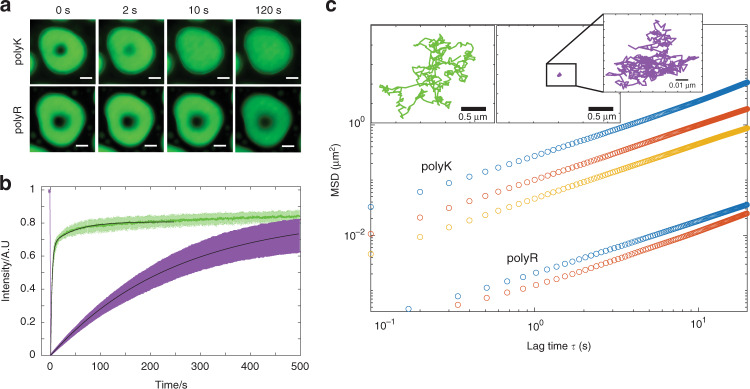
Differences in emergent properties of polyR and polyK droplets. **a** Confocal fluorescence images of FRAP recovery for polyK10/pU10-A488 (upper) and polyR10/pU10-A488 (lower) illustrating increased fluidity of polyK vs polyR. **b** FRAP recovery within droplets of polyK/pU10-A488 (green) and polyR/pU10-A488 (purple). **c** MSD vs lag time for polyK and poly R of length 10 (blue, o), 50 (red, o) and 100 (yellow, o) with UTP) illustrating increased viscosity of polyR compared to polyK. Inset: Brownian motion of 200 nm bead in polyK50/UTP (green) and polyR50/ UTP (purple).

We next sought to determine how differences in droplet stability and interaction strength influence the relative material properties of polyK/polyR droplets. FRAP measurements reveal a dramatic decrease in recovery of pU10 within polyR droplets in comparison to polyK (Fig. 3a). Upon bleaching a spot (radius *r* = 1.5 μm) in the center of droplets containing 1% Alexa 488-labeled pU10 RNA, we find that over the course of approximately 10 s polyK10 droplets recover to approximately 80%, whereas polyR10 droplets recover to this value on the order of 1000 s (Fig. 3b). Using

4 $$D_{{\rm{app}}} = \frac{{r^2}}{t},$$

where *t* is recovery time, we find an apparent diffusion coefficient of 2.18 × 10^−13^ m^2^ s^−1^ for polyK compared to 2.9 × 10^−15^ m^2^ s^−1^, for polyR indicating an approximately 100-fold difference in mobility. To precisely quantify changes in viscosity, we next perform microrheology experiments. We find that polyR droplets are viscous fluids with viscosities ranging from 36 Pa.s for polyR10/UTP to >200 Pa.s for polyR complexed with pU50, translating to approximately 30×–300× higher viscosities (consistency of ketchup) than comparable polyK constructs (consistency of maple syrup)  (Fig. 3c and Table 1). We find that the greatest relative increase in viscosity is seen for the UTP conditions. We note that viscosity differences between sequences of equal length are significantly greater than the relative difference between polymer lengths of a single residue. Interestingly, the viscosities of pure polyK and polyR solutions in the absence of nucleotide-induced phase separation are equivalent up to the highest concentration tested (see Supplementary Table 1 for details). Together, these results highlight a role for distinct modes of nucleotide complexation vs homotypic residue–residue interactions in contributing to the significant differences in viscosity between polyR and polyK droplets.

### R/K differences sufficient to induce multiphase coexistence

Our results thus far indicate that differences in nucleotide interaction strength and interaction modes between polyK and polyR dramatically influence droplet viscosity. We therefore hypothesize that this dramatic difference between polyK and polyR nucleotide interactions could be sufficient to drive the formation of multiphase condensates.

**Fig. 4:**
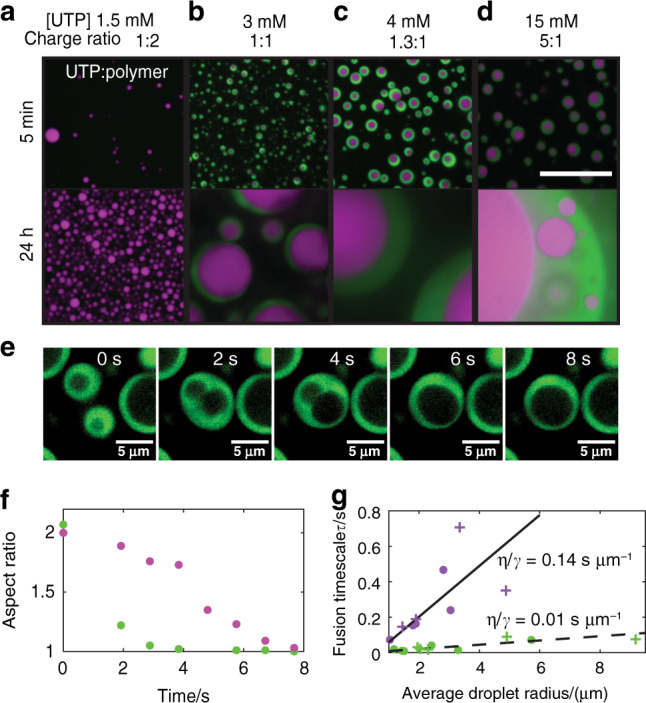
Multiphase condensate behavior. **a**–**d** Confocal fluorescence images of multiphase liquid condensates formed from the addition of UTP (1.5 mM (**a**), 3 mM, 4 mM or 15 mM (**b**–**d**)) to polyK:polyR 50:50 mixtures. Scale bar 20 μm **e** Confocal fluorescence images of fusion of dual-phase coacervates. polyK phase (green) and polyR phase unlabeled. **f** Aspect ratio change outer polyK droplet (green) and inner polyR droplet (purple). **g** Fusion timescale vs average droplet radius for polyR single-phase droplets (purple circles), polyR dual-phase droplets (purple crosses), polyK single-phase droplets (green circles), polyK dual-phase droplets (green crosses).

To investigate this hypothesis, we combined 50:50 mixtures of polyK (6 mM monomer) and polyR (6 mM monomer) with varying amounts of UTP. Indeed, we observe the formation of multiphase droplets in all conditions with sufficient UTP (3 mM) to form charge-matched condensates with both polymers (Fig. 4b–d). Where UTP was present at a concentration at which only half the total polymer mix could form a charge-matched condensate (1.5 mM), we observe a single polyR phase (Fig. 4a). A partition coefficient (P) of approximately 1.2, calculated from fluorescence intensities, indicates that polyK is only slightly enhanced with in this phase compared to polyR (*P* = approximately 11).

Multiphase condensates of all conditions tested form with polyR as the inner layer and polyK as an external shell. This implies that the polyR phase has a higher density than polyK^48^ consistent with maintaining a higher viscosity, as well as a higher surface tension^45^. We find that fusion of the inner polyR droplets occurs more slowly than fusion of the surrounding polyK droplets (Fig. 4e, f and Supplementary Movie 1). To obtain an approximate value of surface tension (*γ*), inverse capillary velocity (*η*/*γ*) can be approximated from the slope of fusion time (*τ*) vs average droplet radius (*l*) by assuming phases are simple liquids in a lower viscosity medium where $\tau \approx l(\eta /\gamma )$^4,6,30,59^. For single-phase pure polyK droplets, we find *η*/*γ* ≈ 0.012 s μm^−1^. Having measured the viscosity directly, we can calculate an approximate surface tension of 17 μN m^−1^. We find that, when multiphase droplets fuse, the polyK component fuses at the same timescale as pure polyK, as illustrated by single and multiphase fusions following the same linear trend (Fig. 4g). For single-phase, pure polyR droplets, we find *η*/*γ* ≈ 0.144 s μm^−1^ and *γ* ≈ 100 μN m^−1^; these values are approximately an order of magnitude higher than those obtained for polyK. Similar to polyK, multiphase and single-phase droplets appear to follow the same *τ* vs *l* linear trend, indicating that polyR most likely retains its highly viscous properties in a multiphase environment. Interestingly, the number of polyR fusion events was found to increase in multiphase droplets apparently due to readily fusing polyK droplets forcing polyR droplets into close proximity.

### polyR antagonizes polyK condensates triggering polyK release

Upon demonstrating that polyR and polyK are capable of forming distinct coexisting phases, but only at sufficiently high UTP concentrations, we next sought to investigate the impact of the order of addition of polyK/polyR solution components. We find that, although the order of addition does not affect the final equilibrium state, the mechanism by which this equilibrium state is reached is dramatically different. For the multiphase conditions, when polyK is added to pre-formed polyR droplets, initially no change is observed but with sufficient time a secondary polyK phase will form surrounding the existing polyR liquid phase (Supplementary Fig. 6 and Supplementary Movies 2–5). As was seen in the premixed samples, only a single polyR phase is observed at limiting UTP concentration (1.5 mM) even at long timescales.

**Fig. 5:**
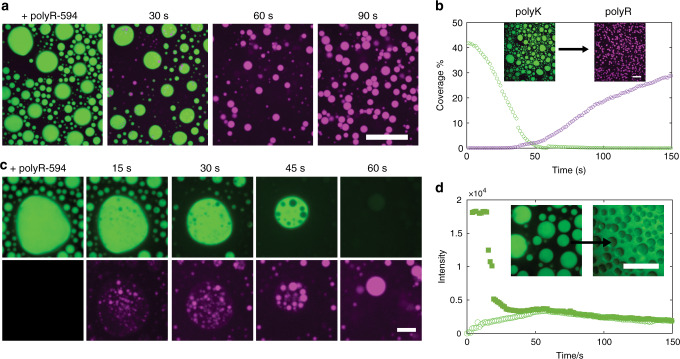
Condensate inversion and polyK release. **a** Confocal fluorescence images of polyK fluorescein isothiocyanate (FITC)-labeled (green) displacement by polyR50 labeled with dylight594 (purple). Merged images taken at moment of polyR addition (*t* = 0) and after 30, 60, and 90 s. Scale bar 20 μm. **b** Percentage of slide covered as a function of time for polyK (green) and polyR (purple). Inset images correspond to *t* = 0 and *t* = 90 s. Scale Bar 20 μm. **c** Close up of individual condensate green channel showing polyK FITC and purple channel only showing polyR50. Scale bar 5 μm. **d** Intensity of FITC-polyK over time inside a polyK droplet (filled square) and outside of a polyK droplet (open circle). Intensity values correspond to timeseries displayed in **a**. Inset displays polyK FITC before and after displacement by unlabeled polyR50 illustrating polyK displacement into surrounding media. Scale Bar 20 μm Intensity re-scaled in this image for clarity. [polyK] = [polyR] = 6 mM monomer. [UTP] = 1.5 mM.

More remarkably, however, when we first form charge-matched polyK-UTP condensates (6 mM polyK/1.5 mM UTP) and subsequently add an equal amount of polyR50, within around 60 s we observe complete condensate inversion with polyK droplets being entirely replaced by polyR (Fig. 5a, b). Zooming in on individual droplets (Fig. 5c), we find that polyR50 nucleates droplets within polyK-rich condensates; monitoring the polyK fluorescence, we see that polyR50 nucleation is concomitant with polyK release from the condensate to the surrounding environment (Fig. 5d). This illustrates that polyR is successfully competing for the available UTP, thereby triggering the release of free polyK back to the dilute phase. When the experiment is repeated with polyR100, which assembles into amorphous aggregates in the presence of UTP (Fig. 2a), we find that polyK is ultimately released after the droplets transform to aggregates (Supplementary Fig. 6), further demonstrating the dominance of polyR over polyK interactions.

### Manipulating release kinetics and coexisting liquid phases

Triggering the rapid release of a component in a condensed phase presents a useful tool for engineering droplet dynamics. We next asked whether tuning our minimal model components could regulate the kinetics of multiphase dynamics.

**Fig. 6:**
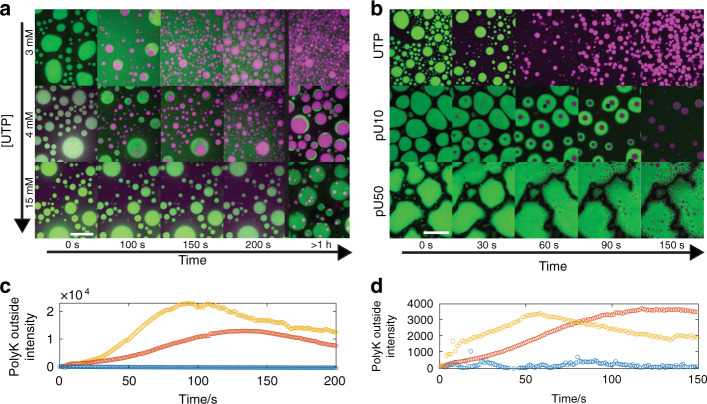
Control over inversion and multiphase coacervate creation. **a** Confocal fluorescence images of droplet inversion via addition of Dylight-labeled polyR50 at increasing UTP concentrations (3, 4, 15 mM top to bottom). Timepoints 0, 100, 150, 200 s, and 1 h are shown. Scale bar = 20 μm. **b** Initial coacervates of polyK paired with (i) UTP, (ii) pU10, and (iii) pU50. Scale bar = 20 μm. **c** Intensity of FITC-labeled polyK in dilute phase for UTP concentrations 3 mM (red), 4 mM (gold), and 15 mM (blue). Intensity values correspond to timeseries displayed in **a**. **d** Intensity of FITC-labeled polyK in dilute phase for UTP (gold), pU10 (red), and pU50 (blue). Intensity values correspond to timeseries displayed in **b**.

We had previously shown that addition of polyR50 to polyK droplets at limiting UTP concentrations (1.5 mM) resulted in rapid inversion and release of polyK to the dilute phase (Fig. 5), consistent with the single polyR phase present in our equilibrium experiments (Fig. 4). Given that our equilibrium experiments indicate the presence of multiphase droplets at higher UTP concentrations, we hypothesized that increasing the relative UTP abundance would in turn impact the inversion dynamics. Indeed, we find that increasing the UTP concentration controls the kinetics of polyK release as well as the stabilization of coexisting phases over long timescales (Fig. 6a, c and Supplementary Movies 6–9). We find that, at 3 and 4 mM UTP, inversion still occurs but at increasing timescales within approximately 2 and 5 min, respectively, compared to approximately 1 min for 1.5 mM (Fig. 5a) with no observable polyK release at 15 mM UTP. Interestingly, for 3 mM UTP and more significantly for 4 mM UTP, we find that, after rapid release of polyK to the dilute phase, a polyK-rich phase begins to re-condense around the polyR phase on the timescale of hours. These dual-phase droplets persist up to at least 24 h (Supplementary Fig. 5), resembling the equilibrium state described above.

Given the demonstrated impact of polymer length on droplet viscosity (Table 1 and Fig. 3), we then asked whether polymer length could also be used to tune intra-droplet dynamics. We repeated the inversion experiment and compared UTP, pU10, and pU50, all under initial charge-matched conditions (Fig. 6b, d, Supplementary Movies 10 and 11). Indeed, we find that pU10 and pU50 progressively dampen polyK release kinetics along with increasing the longevity of coexisting phases. Thus tuning either the stoichiometry or the polymer length of droplet components can regulate the dynamics of this multiphase model system.

## Discussion

As the broader impact of liquid phase separation on the fields of cell biology and bioengineering continues to expand, many fundamental questions and challenges remain. For example, how do sequence-level changes influence condensate material properties; how do material properties in turn influence condensate dynamics, multiphase coexistence, and ultimately function; and finally, can bottom–up sequence design rules be generated to engineer condensates with specific material properties that can be leveraged for controlling condensate dynamics.

The molecular and functional differences between arginine and lysine residues make them an ideal model system for extracting fundamental principles bridging sequence, material, and functional levels. In addition to unique roles in liquid phase separation discussed above, arginine specificity over lysine residues appear in other functional roles, including membrane interactions^60^, cell entry of antimicrobial peptides^61^, and regulation of voltage-gated ion channels^62^. In addition, both arginine and lysines and their respective post-translational modifications play important roles in chromatin remodeling via regulation of histone proteins^63^. While both lysine and arginine have the same theoretical positive charge at neutral pH (pKa’s ~10.5 and 13.8 for lysine and arginine, respectively), the delocalization of positive charge within the pi-bonded guanidium side chain of arginine imparts it with enhanced modes of interactions. While both cations can engage in cation–pi interactions, arginine alone can further engage in pi–pi interactions^25,28,33^ via its guanidium group as well as hydrogen bonding^64^. It is worth noting that, while we cannot rule out any local pKa shifts in lysine vs arginine within coacervates that influence effective charge, such shifts would not likely support the drastic differences we see in our viscosity measurements. Arginines are also more efficient at RNA binding^65^, and recent work^40^ suggests that proline–arginine dipeptide repeats interact more strongly with RNA oligonucleotides than proline–lysine repeats due in part to enhanced pi–pi interactions. This is consistent with our phase diagram and FCS binding data, which show significant increase in interaction strength for polyR compared to polyK with respect to pU binding. In addition to enhanced interaction strength, the delocalization of charge on the arginine group may contribute to enhanced effective multivalency, both of which would be consistent with the significant increases in emergent viscosity we report here (Fig. 3). This additionally aligns with our divergent results for assembly capacity of polyR/polyK with UDP and UTP, whereby enhanced nucleotide interactions enable only polyR to form droplets with UDP and leads to the aggregation of polyR100-UTP (Fig. 2).

The same difference in molecular interaction strength that leads to distinct viscosities also drives the assembly of multilayered condensates. Both multiphase inversion and coexistence can be understood in terms of the relative ability of polyR and polyK to compete for UTP binding. Jacobs and Frenkel showed theoretically that for systems where sufficient interaction strength differences between components exists multiple distinct phases will form^66^. Interestingly, they also show that, as the number of components increases, the system tends toward forming a single phase. It is therefore important to bear in mind that in vitro systems with few components may tend toward multiple phases at a lower interaction difference than would actually be found in the complex multi-component cellular environment. Experimentally both Mountain and Keating and Lu and Spruijt^48,49^ showed for a three-component system, say two polycations and a single polyanion, that, where some sufficient interaction strength difference between polycations exists, two phases will form with the shared oppositely charged polymer unequally distributed between the two phases based on relative interaction strength. Lu and Spruijt calculate the magnitude of this sufficient difference from density, which they extrapolate from a difference in critical salt concentration^49^. We show that where UTP is the limiting component this uneven distribution of the shared component is so extreme that only polyR phase will form and it is only when UTP is in excess that multiphase formation is observed.

We believe the UTP concentration-dependent inversion kinetics likely results from a differential concentration gradient between the inside and outside of condensates as well as local competition between polyK and polyR for UTP binding. Complex coacervates are generally considered to form between charge-matched quantities of polyanion and cation; consequently, as overall UTP concentration increases but polymer concentration remains the same, the relative external UTP concentration will increase and one would expect to find that the driving force to nucleate within the PK droplets will decrease (Fig. 6). If we consider droplet inversion in terms of competitive binding where polyK + UTP $\rightleftharpoons$ polyK-UTP, polyR + UTP $\rightleftharpoons$ polyR-UTP, and polyK-UTP + polyR $\rightleftharpoons$ polyK + polyR-UTP, both the rate of polyK-UTP dissociation and polyR UTP binding will determine the rate of polyR/UTP droplet formation. At increasing UTP concentration, the rate of dissociation should not be affected. As polyanion length increases, however, due to increased number of interaction sites per chain, a slower rate of dissociation could be expected, accounting for the observed increase in inversion time with increasing polyanion length. We additionally note the observation of the formation of vacuoles, presumed to consist of surrounding buffer, during the dynamics of inversion in pU10 droplets (Fig. 6b) and even more so in pU50 droplets, but not in UTP samples, suggesting increasing prevalence with increasing viscosity. This non-equilibrium vacuole formation has been observed previously in biomolecular condensates and has been shown to be capable of being induced in polyK DNA systems by exposure to an electric field^45,67,68^. We have not observed vacuole formation when polyK and polyR are premixed indicating that their formation is not favorable under equilibrium conditions and only occur during the disruptive process of polyK droplet disassembly.

Here we have demonstrated bottom–up control of multi-droplet assembly and dynamics by exploiting the contribution of polymer length, stoichiometry, and the distinct differences between lysine and arginine residues and their respective nucleotide interactions. Employing minimalist polymers and precise rheological measurements, we have dissected the contribution of arginine/lysine residues to the bulk material properties of biomolecular liquid condensates. We demonstrate that the distinct modes of arginine/lysine interactions with mononucleotides and polynucleotides gives rise to individual droplets with viscosities that differ by orders of magnitude, which can be finer tuned by polymer length. Arginine and lysine polymers are not miscible within condensates, with arginine outcompeting lysine for anionic partners. Importantly, we go on to show that the fundamental differences in arginine/lysine–nucleotide phase behavior can be exploited to trigger the controlled release of lysine sequences and to drive the formation of coexisting immiscible phases with tunable kinetics of self-separation. Together, this work lends unique insight into the distinct roles of arginines and lysines in liquid phase separation and more significantly provides fundamental design principles for leveraging sequence-level components in order to regulate droplet assembly, dynamics, and multiphase coexistence. These principles present invaluable tools for the regulation and engineering of novel organelles and could feasibly be developed into incorporating lysine/arginine tags designed to modulate molecular release and phase behavior with tunable kinetics. Expanding this fundamental model toward increased sequence complexity, component diversity, and post-translational modifications presents exciting new future directions.

## Methods

### Materials

Poly(L-lysine hydrochloride) (molecular weight (MW) = 1600 Da, *N* = 10, DP_*n*_ by nuclear magnetic resonance (NMR) = 8–12), poly(L-lysine hydrochloride) (MW = 8200 Da, *N* = 50, DP_*n*_ by NMR = 45–55), poly(L-lysine hydrochloride) (MW = 16 kDa, *N* = 100, DP_*n*_ by NMR = 90–110), poly(L-arginine hydrochloride) (MW=1900 Da, *N* = 10, DP_*n*_ by NMR = 8–12), poly(L-arginine hydrochloride) (MW = 9600 Da, *N* = 50, DP_*n*_ by NMR = 45–55), and poly(L-arginine hydrochloride) (MW = 19 kDa, *N* = 100, DP_*n*_ by NMR = 90–110) were purchased from Alamanda Polymers (Huntsville, AL, USA) and used as received. Poly(L-lysine hydrochloride) (MW = 15–30 kDa) and poly(L-lysine hydrochloride)–fluorescein isothiocyanate (FITC) labeled (MW = 15–30 kDa) were purchased from Sigma-Aldrich. Polymer stock solutions (50 mg ml^−1^) were prepared in nuclease-free water and stored at 4 °C. Solutions were sonicated for 10 min, as per the manufacturer’s instructions, and diluted in Tris buffer prior to use. UTP and UDP were purchased from MP Biomedicals (Solon, OH, USA). UMP was purchased from Sigma. pU RNAs (*N* = 10 and *N* = 50) were purchased from IDT. pU RNAs were received as lyophilized samples that were resuspended in TE buffer (10 mM Tris pH 8.0, 0.1 mM EDTA) at 1 mM and stored at −20 °C. UTP, UDP, and UMP were prepared in nuclease-free water (90 mM) and stored at 4 °C for immediate use or at −20 °C for longer-term storage.

### Poly-L-arginine labeling

Poly(L-arginine hydrochloride) (*N* = 50) was labeled with Dylight594 via amide linkage as per the manufacturer’s instructions. Unreacted dye was removed using a Hi-trap de-salting column, equilibrated with Tris (10 mM, pH 7.5), and connected to an AKTAstart, followed by overnight dialysis (3 kDa cut off) in the same buffer. Concentration was determined from FCS measurements.

### Coacervate preparation

Coacervate samples were prepared by the addition of charge-matched quantities of polyanion to solutions of polycation (6 mM monomer). Charge matching was assumed to be a 4:1 ratio for UTP, 3:1 for UDP, and 1:1 per nucleotide monomer of pU RNA. For microrheology samples, 200 or 500 nm beads were added prior to the addition of polyanion. Samples were imaged in glass slide–coverslip chambers made with Grace BioLabs spacers. To prevent the droplets wetting the surface of the well, slides were first incubated in a 1% Pluronics F-127 solution for 1 h followed by thoroughly rinsing with MilliQ water.

For multiphase complex coacervate experiments, samples were prepared in Grace BioLabs CultureWells with coverslip bottom and treated with 1% Pluronics F-127 (1 h).

### Coacervate imaging

Samples were imaged on a Marianas Spinning Disk confocal microscope (Intelligent Imaging Innovations) consisting of a spinning disk confocal head (CSU-X1, Yokagawa) on a Zeiss Axio Observer inverted microscope equipped with ×100/1.46 numerical aperture (NA) Plan-Apochromat (oil immersion) or ×63/1.4 NA Plan-Apochromat (oil immersion). Focus was maintained over time using Definite Focus 2 (Zeiss). FITC or Alexa488 were excited with the 488-nm line from a solid state laser (LaserStack) and collected with a 440/521/607/700-nm quad emission dichroic and 525/30-nm emission filter. Dylight and carboxy-labeled beads were excited with the 561-nm line and collected with the same dichroic and 617/73-nm emission filter. Images were acquired with a Prime sCMOS camera (Photometrics) controlled by SlideBook 6 (Intelligent Imaging Innovations). ImageJ was used to further format and process images. Images of Dylite594-labeled polyR have been false colored purple to improve contrast.

### Phase diagrams

Phase diagrams were constructed by brightfield imaging of polymer/UTP/salt mixtures prepared in glass slide–coverslip chambers. Imaging was performed approximately 30 min after mixing using a ×63 objective on an inverted Zeiss Axio microscope. Based on these observations, samples were designated as either droplet or no droplet.

### Fluorescence recovery after photobleaching

FRAP experiments were performed on a Marianas Spinning Disk confocal microscope with a ×63/1.4 NA Plan-Apochromat oil immersion objective. An area with radius = 1.5 μm was bleached with a 488-nm laser; subsequent recovery of the bleached area was recorded with a 488-nm laser. Intensity traces were exported from Slidebook (Intelligent Imaging Innovations, Denver, CO). Correction for photobleaching, normalization, and fitting to an exponential function of the form

5 $$f\left( t \right) = A\left( {1 - e^{\frac{{ - t}}{\tau }}} \right)$$

was performed in Matlab. The final FRAP recovery curve is the average of recovery curves collected from five separate droplets.

### Microrheology

Fluorescent beads of 100, 200, or 500 nm were embedded into droplets typically >50 μm and their motion was tracked over time to obtain the MSD. To avoid boundary effects, only beads several microns away from all droplet interfaces were analyzed. Bead diffusion was tracked on a Marianas Spinning Disk confocal microscope with a ×100/1.46 NA Plan-Apochromat oil immersion objective for 1000 frames with 100, 200, or 500-ms time intervals. Temperature was kept at 19 °C using a microfluidic temperature stage (CherryTemp, CherryBiotech). Particle-tracking code to locate and track bead trajectories in two dimension (*XY*) was adapted from Matlab Multiple Particle Tracking Code from The Matlab Particle Tracking Code Repository (http://physics.georgetown.edu/matlab/index.html). Custom Matlab software was then used to analyze bead dynamics.

MSD was calculated from time and ensemble averages for all trajectories:

6 $${\rm{MSD}}\left( \tau \right) = \left\langle {\left( {x\left( {\tau + t} \right) - x(t)} \right)^2} \right\rangle + \left\langle {\left( {y\left( {\tau + t} \right) - y(t)} \right)^2} \right\rangle.$$

The dependence of the MSD on lag time (*τ*) follows a power law, the exponent ∝ was determined as the slope of a log–log plot and diffusion coefficient as the *y*-intercept.

7 $${\rm{MSD}}\left( \tau \right) = 2dD\tau ^ \propto,$$

where *d* is the number of dimensions (here 2), *D* is the diffusion coefficient, and ∝ is the exponent.

Viscosity can be determined from the Stokes–Einstein relation (Eq. 3), assuming a system at equilibrium and a freely diffusing Brownian particle within a solution of viscosity *η*.

The final viscosity is the average of three values collected from individual measurements performed on 3 different days at 19 ± 2 °C. Errors shown are standard deviations derived from these three individual experiments.

### Inversion experiments

Poly(L-lysine hydrochloride) (MW = 15–30 kDa, 10% labeled) and UTP coacervates were allowed to sit for 2 h at which point droplet settling had subsided. An equal amount of poly-L-arginine 50 (approximately 1% labeled; [polyK] = [polyR]) was then added as a timeseries was recorded. Timeseries were recorded with a time interval approximately 1 s. *t* = 0 was assigned as the first frame emission was detected in the red channel. Intensity plots (Figs. 5c and 6c, d) are representative plots from a single timeseries, not averages of multiple runs. The average inversion time from three measurements is shown (Supplementary Fig. 7).

### Fusion experiments

For fusion measurement, samples were prepared in Grace BioLabs CultureWells with coverslip bottom treated with 1% Pluronics F-127 (1 h). Timeseries of fusion events were collected at 49-ms time intervals on a widefield Axio Observer 7 Inverted Microscope (Zeiss) with a ×63/1.4 NA Plan-Apochromat (oil immersion) objective. FITC was excited with a 120-W metal halide lamp (X-cite 120) with 470/40 nm excitation and 525/50 nm emission filters. Images were acquired with a Axiocam 506 mono camera (Zeiss) controlled by the Zen software (Zeiss). ImageJ was used to further format and process images, and MATLAB was used to analyze fusion events as described previously^69^.

### Fluorescence correlation spectroscopy

FCS binding measurements were performed on an inverted Leica TCS-SP8 STED 3X equipped with a ×63 water immersion objective. Fluorophores were excited at 488 nm using a white light laser and detected at 550 ± 20 nm using an HyD detector. Temperature was kept constant at 25 °C using a temperature stage (Instec Inc., CO, USA, Model mK2000B). Data acquisition and calculation of the correlation curve *G*(*τ*) were performed with the SymPhoTime software (PicoQuant, Germany). Each measurement is the average of ten 30 s traces. Averaged autocorrelation curves were then fit to a single-component model using the following equation:

8 $$G\left( \tau \right) = \frac{1}{{\left[N\left( {1 + \frac{\tau }{{\tau _{\rm{D}}}}} \right)\left( {1 + \frac{\tau }{{{\it{\upkappa }}^2\tau _{\rm{D}}}}} \right)^{0.5}\right]}},$$

where *G*(*τ*) is the autocorrelation function as a function of time, *τ*. *N* is average number of molecules in the focal volume. *τ*_D_ is diffusion time, the average amount of time a molecule spends diffusing through the observation volume. ${\it{\upkappa }} = \frac{{z_0}}{{\omega _0}}$ represents the ratio of the axial (*z*_0_) to radial (*ω*_0_) dimensions of the Gaussian excitation volume. This value was determined by calibration using Atto488-carboxylic acid (*D* = 4.0 × 10^−6^ cm^2^ s^−1^ at 25 °C).

## Supplementary information

Supplementary Information

Description of Additional Supplementary Files

Supplementary Movie 1

Supplementary Movie 2

Supplementary Movie 3

Supplementary Movie 4

Supplementary Movie 5

Supplementary Movie 6

Supplementary Movie 7

Supplementary Movie 8

Supplementary Movie 9

Supplementary Movie 10

Supplementary Movie 11

## Data Availability

The data sets generated during and/or analyzed during the current study are available from the corresponding author on reasonable request.
