# Supplementary material for: Tunable multiphase dynamics of arginine and lysine liquid condensates
Source: Nat Commun. 2020 Sep 15;11:4628. doi: 10.1038/s41467-020-18224-y (PMC7492283; doi:10.1038/s41467-020-18224-y)
Supplement: Supplementary file 1 — Supplementary Information [file 41467_2020_18224_MOESM1_ESM.pdf]

Supplementary Information

Tuning multiphase dynamics of arginine and lysine liquid condensates.

Rachel S. Fisher[1], Shana Elbaum-Garfinkle[1,2]
[1]Structural Biology Initiative, CUNY Advanced Science Research Center, New York, NY, United States
[2]Ph.D. Programs in Biochemistry and Biology at the Graduate Center, CUNY, New York, NY, United States

Supplementary Figures

Supplementary Figure 1. Poly-lysine Coacervates. Coacervation conditions tested for poly-lysine with UMP, UDP, UTP, pU10 and pU50. No coacervates were observed for poly-lysine (6mM) with charge matched concentrations of UMP (3 mM) or UDP (2 mM) but coacervates were observed for UTP (1.5 mM), pU10 (6mM) and pU50 (6mM). (All concentrations are per monomer). Scale bar = 20 µm.

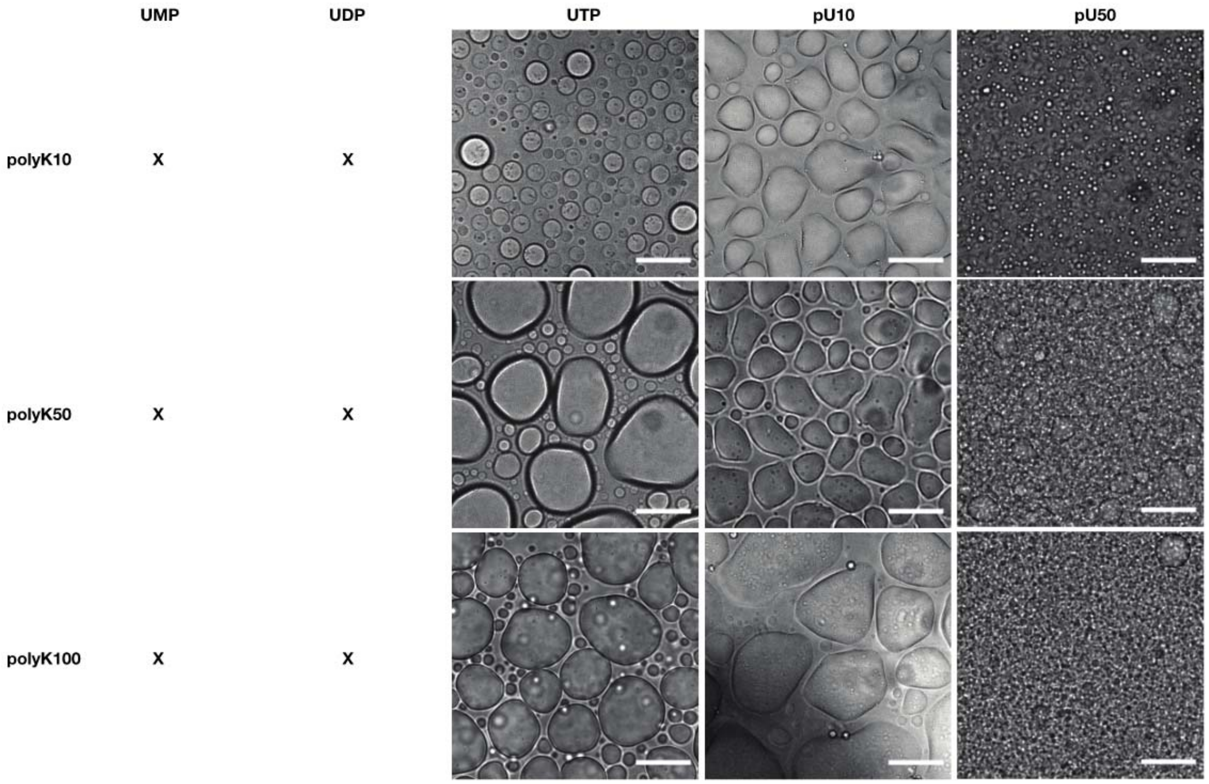

Supplementary Figure 2. Loglog plot of viscosity as a function of PolyK polymer length. Exponent of 1 for pU10 (purple) and pU50 (blue), 0.7 for UTP (red). Points shown are mean ± SD for n = 3 individual measurements.

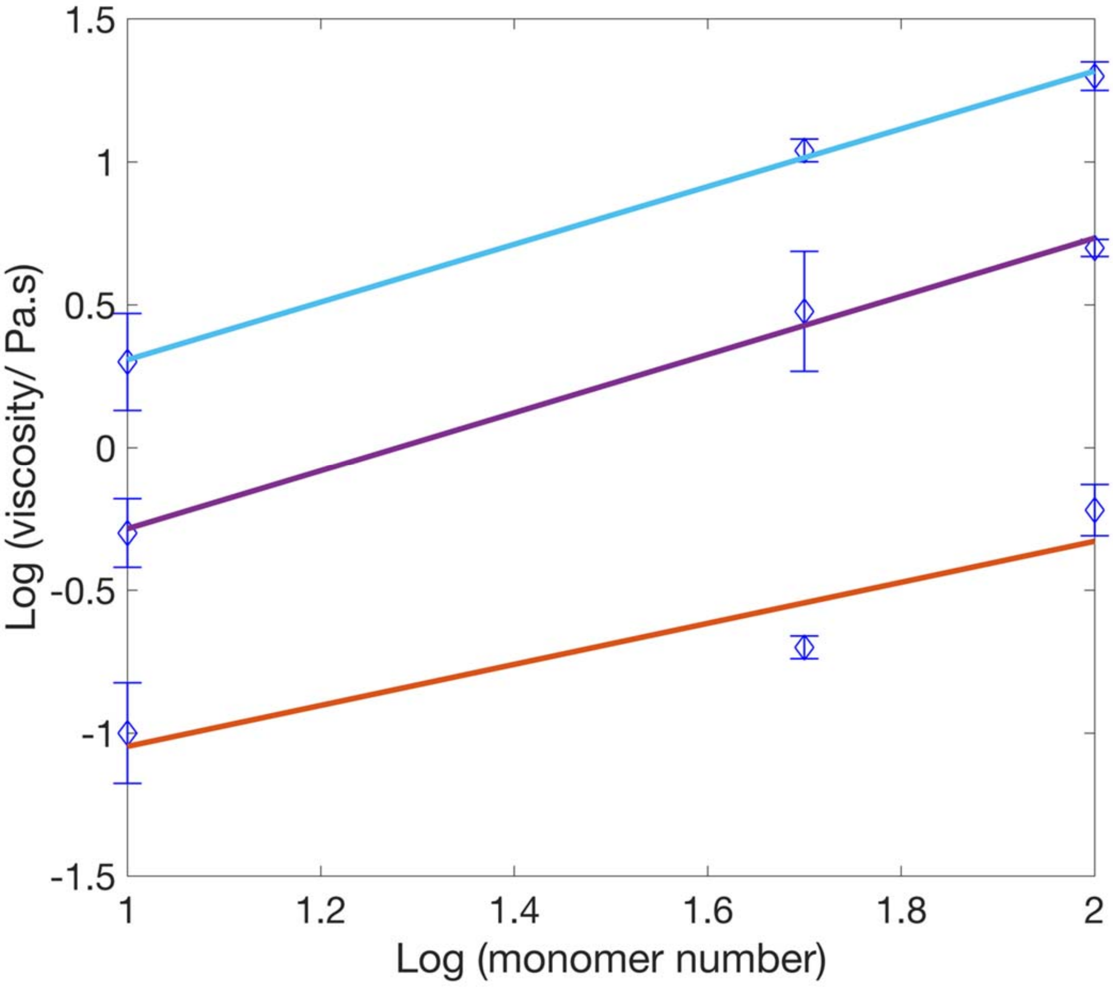

Supplementary Figure 3. Poly-arginine Coacervates. Coacervation conditions tested for poly-arginine with UMP, UDP, UTP, pU10 and pU50. No coacervates were observed for poly-arginine (6mM) with charge matched concentrations of UMP (3 mM) but coacervates were observed for UDP (2mM) UTP (1.5 mM), pU10 and pU50. Scale bar = 20 μm.

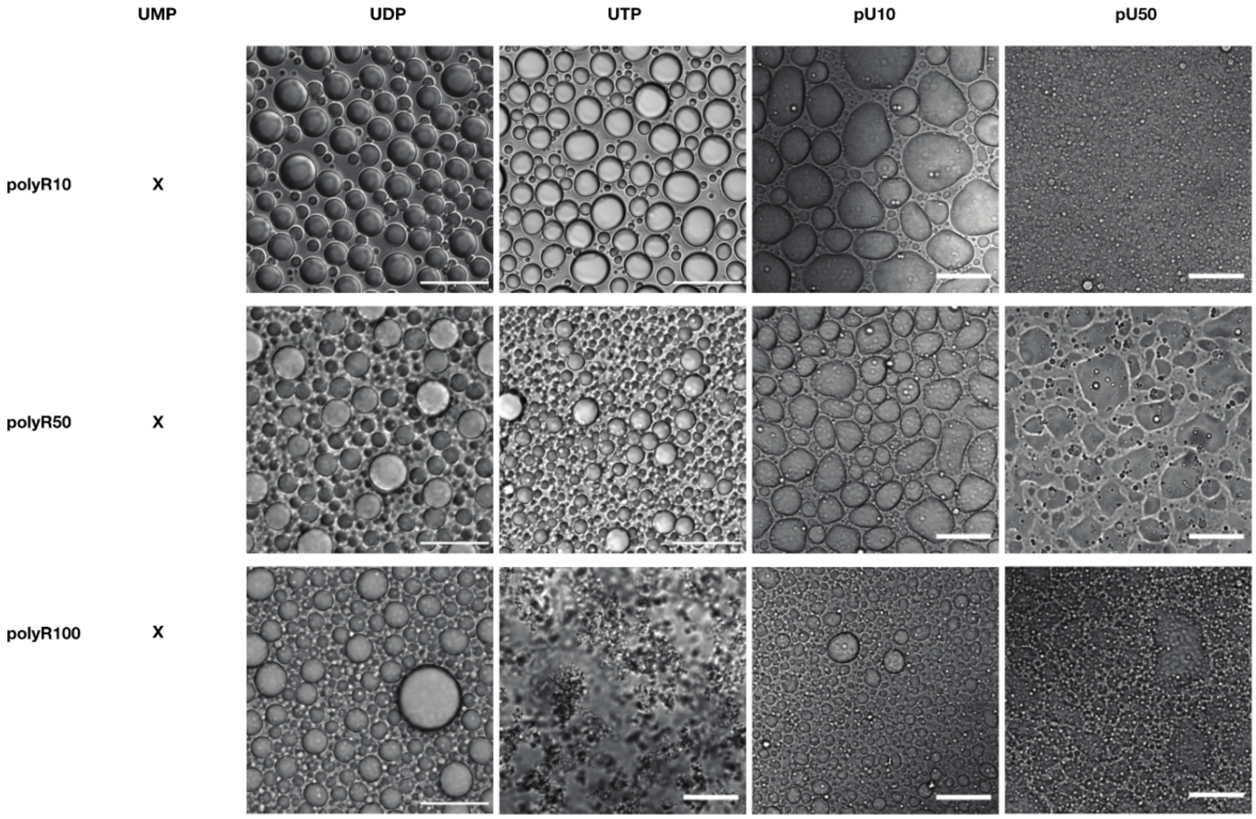

Supplementary Figure 4. PolyK vs PolyR binding affinity. PolyK (green) and polyR (purple) binding to pU10-Alexa488 as measured by fluorescence correlation spectroscopy. Plot of diffusion time (τ_D) of pU10 as a function of polymer concentration. Green circles represent mean values ± SD for n = 3 measurements. Remaining points are the result of individual measurements with each symbol representing an individual titration. For polyK binding to pU10-Alexa488 the resulting data was fitted to a four-parameter logistic curve: $f(x) = a + \frac{d-a}{1+(\frac{x}{c})^b}$ where *a* is the minimum diffusion time, *d* is the maximum diffusion time, *b* is the Hill Slope and *c* is the inflection point or concentration at 50% maximum binding capacity.

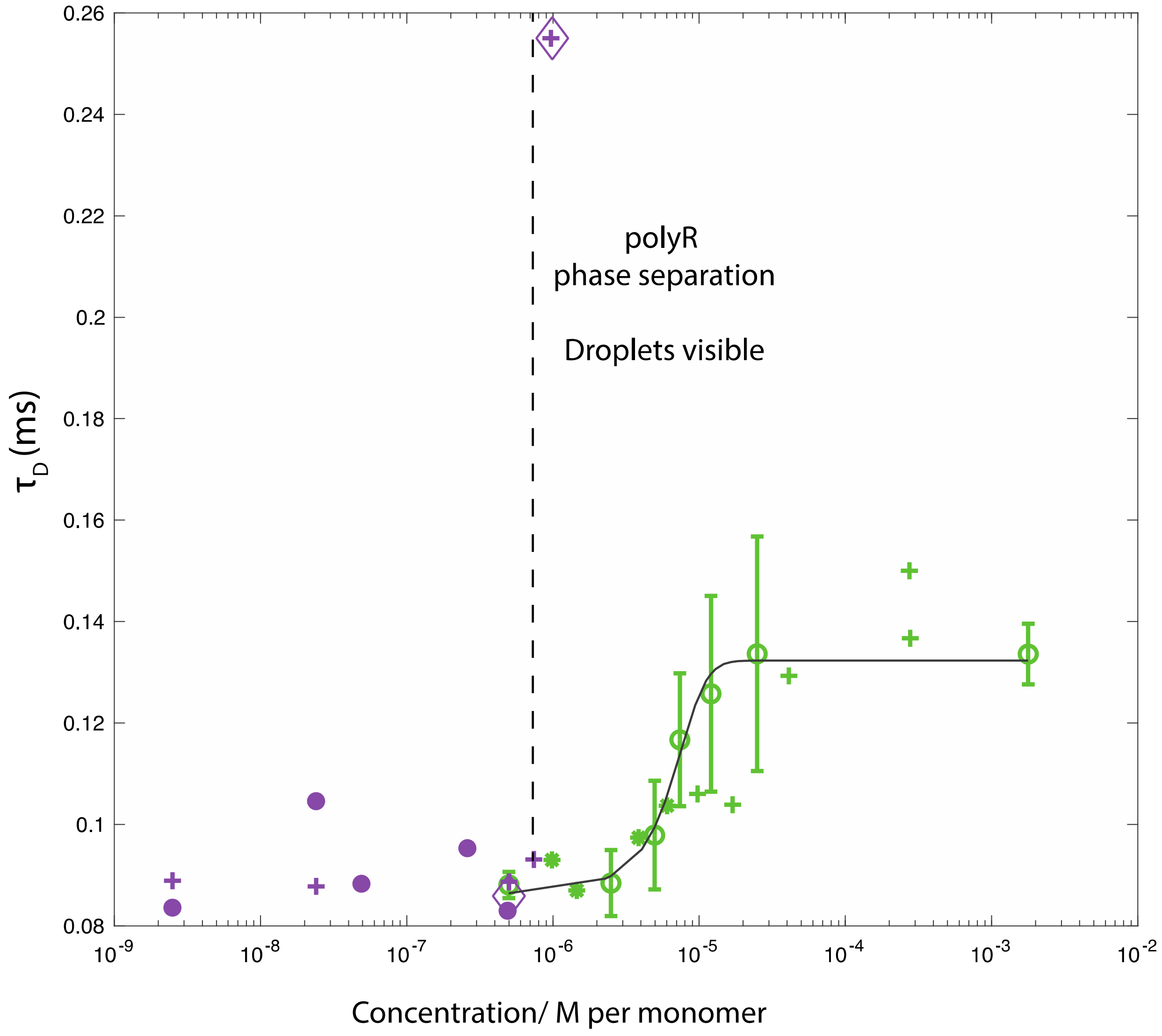

Supplementary Figure 5. Equilibrium droplet state. Top Panel. Droplet inversion via addition of Dylight labelled polyR50 at increasing UTP concentrations (1.5 mM, 3 mM, 4 mM, 15 mM). Images recorded after 24 hours. Middle Panel. Mixed polyR and polyK after 5 mins, 1 hour or 24 hours. Bottom panel. Addition of polyK to polyR50 condensates after 1 hour or 24 hours. Scale bar = 20 μm.

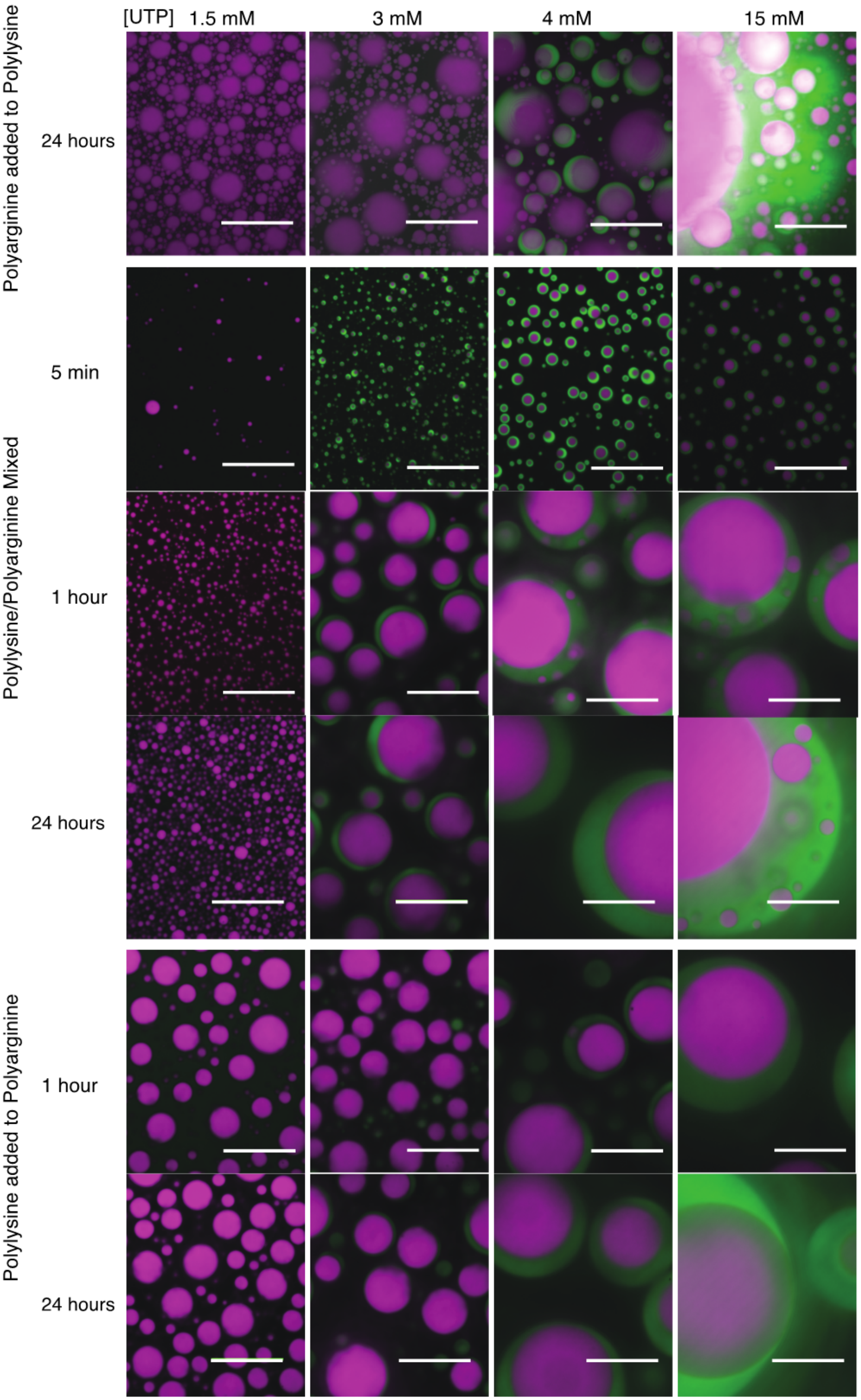

[UTP]
1.5 mM
3 mM
4 mM
15 mM
Polyarginine added to Polylysine
24 hours
Polylysine/Polyarginine Mixed
5 min
1 hour
24 hours
Polylysine added to Polyarginine
1 hour
24 hours

Supplementary Figure 6. PolyK-FITC (green) displacement by polyR100 (unlabeled). Images taken at moment of PR addition (t=0) and after 7,12 and 17 seconds. After 30 seconds condensates have become amorphous aggregates. Scale bar = 20 µm.

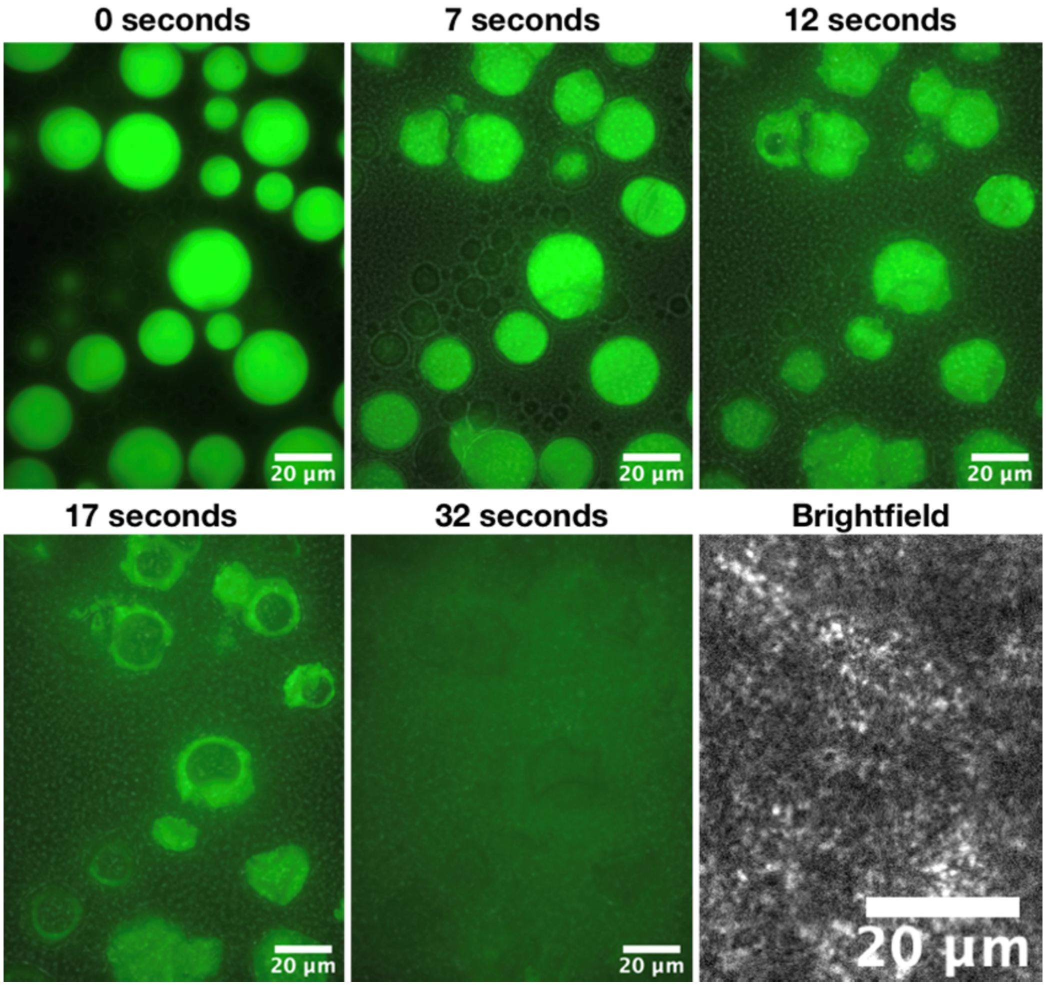

Supplementary Figure 7. Mean inversion times. Bar graph showing mean inversion time (determined from last frame where polyK observable) from n = 3 individual measurements. Time series were recorded for 10 minutes. In all three measurements at 15 mM total inversion had not taken place at this time. b. Snapshots from 100 s at UTP concentrations 1.5 mM, 3mM, 4mM and 15 mM. Scale bar = 20 µm.

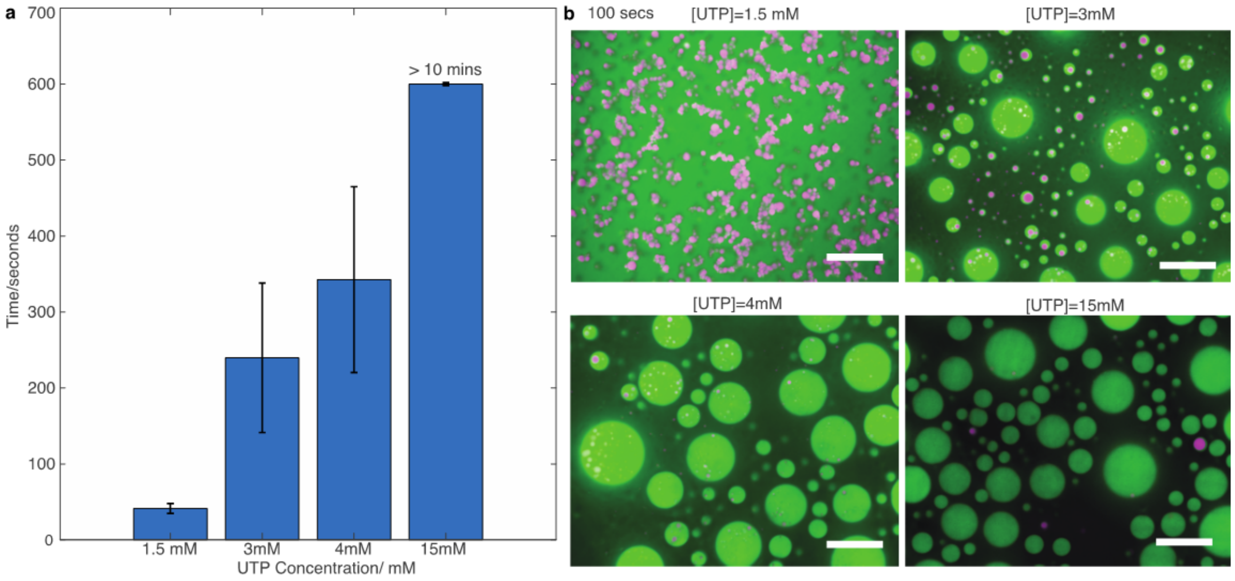

Supplementary Figure 8. Pegylated and non-pegylated beads report the same viscosity. MSD's measured in PolyR10-UTP coacervates.

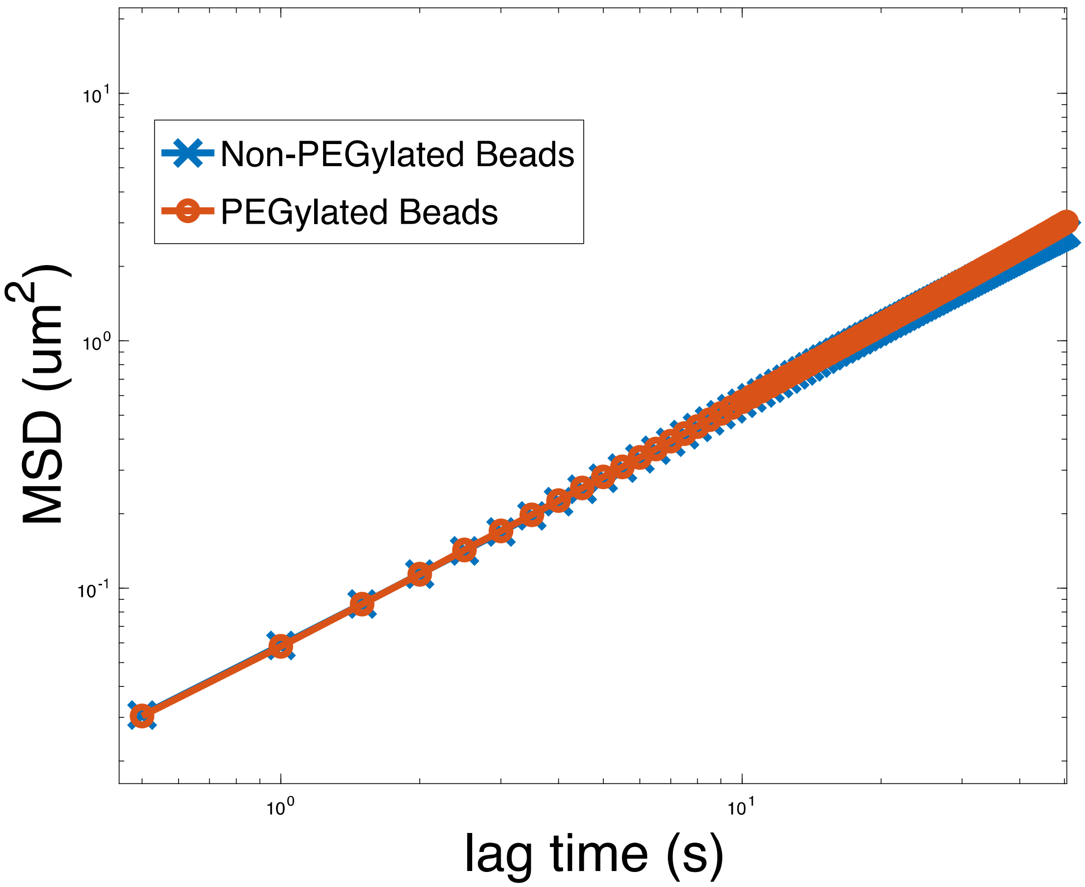

Supplementary Tables

Supplementary Table 1: **Viscosity of polyR and polyK (N=100) in solution** (Tris, 10 mM, pH 7.4) from 2 to 49.5 mg/ml.

PolyR100 Concentration mg/ml	Viscosity / Pa.s	PolyK100 Concentration mg/ml	Viscosity / Pa.s
49.5	0.0027	49.5	0.0036
40	0.0026	40	0.0030
30	0.0021	30	0.0025
20	0.0018	20	0.0020
10	0.0013	10	0.0014
5	0.0012	5	0.0011
2	9.88E-04	2	9.13E-04

Supplementary Methods

Carboxylated microspheres for microrheology: Red carboxylated fluorescent microspheres (FluoSpheres, Life Technologies Corporation) were used as received from the manufacturer. As

a control microspheres (100 nm) were pegylated as follows. PEG-amine (M_r 2K, Rapp Polymere) 5 mg/ ml in MES buffer (50 mM, pH 6) was added at a 1:1 ratio to a 2% solution of microspheres. After 15 minutes of incubation 1-ethyl-3-(3-dimethylaminopropyl)-carbodiimide (EDAC) was added (final concentration, 4 mg/mL). The pH was adjusted to approximately 6.5 using 10 mM NaOH. Following overnight incubation, the reaction was quenched by addition of glycine (final concentration 100 mM). Microspheres where centrifuged at 10000 g for 15 mins, sonicated and washed with PBS (50 mM) 4 times. To test reaction efficiency, pegylated beads were incubated for 1 hour with FITC-BSA (1mg/ml) and then washed three times. Comparison was made to non-pegylated beads incubated with FITC-BSA for the same time. MSDs obtained from pegylated and non-pegylated beads are displayed in Supplementary Figure 8.
