# Supplementary material for: Tunable multiphase dynamics of arginine and lysine liquid condensates
Source: Nat Commun. 2020 Sep 15;11:4628. doi: 10.1038/s41467-020-18224-y (PMC7492283; doi:10.1038/s41467-020-18224-y)
Supplement: Supplementary file 2 — Description of Additional Supplementary Files [file 41467_2020_18224_MOESM2_ESM.pdf]

Description of Additional Supplementary Files

File name: Supplementary Movie 1
Description: Fusion of dual phase condensates. PolyK phase (white), PolyR phase unlabeled.

File name: Supplementary Movie 2
Description: Droplet inversion via addition of polyK-FITC to Dylight labelled polyR50 at UTP concentration 1.5 mM.

File name: Supplementary Movie 3
Description: Droplet inversion via addition of polyK-FITC to Dylight labelled polyR50 at UTP concentration 3 mM.

File name: Supplementary Movie 4
Description: Droplet inversion via addition of polyK-FITC to Dylight labelled polyR50 at UTP concentration 4 mM.

File name: Supplementary Movie 5
Description: Droplet inversion via addition polyK-FITC to Dylight labelled polyR50 at UTP concentration 15mM

File name: Supplementary Movie 6
Description: Droplet inversion via addition of Dylight labelled PolyR50 at UTP concentration 1.5 mM.

File name: Supplementary Movie 7
Description: Droplet inversion via addition of Dylight labelled PolyR50 at UTP concentration 3 mM.

File name: Supplementary Movie 8
Description: Droplet inversion via addition of Dylight labelled PolyR50 at UTP concentration 4 mM.

File name: Supplementary Movie 9
Description: Droplet inversion via addition of Dylight labelled PolyR50 at UTP concentration 15 mM.

File name: Supplementary Movie 10
Description: Droplet inversion via addition of Dylight labelled PolyR50 at pU10 concentration 1.5 mM.

File name: Supplementary Movie 11
Description: Droplet inversion via addition of Dylight labelled PolyR50 at pU50 concentration 1.5 mM.
